# Modeling of a viscoelastic damper and its application in structural control

**DOI:** 10.1371/journal.pone.0176480

**Published:** 2017-06-01

**Authors:** M. H. Mehrabi, Meldi Suhatril, Zainah Ibrahim, S. S. Ghodsi, Hamed Khatibi

**Affiliations:** Department of Civil Engineering, University of Malaya, Kuala Lumpur, Malaysia; Beihang University, CHINA

## Abstract

Conventional seismic rehabilitation methods may not be suitable for some buildings owing to their high cost and time-consuming foundation work. In recent years, viscoelastic dampers (VEDs) have been widely used in many mid- and high-rise buildings. This study introduces a viscoelastic passive control system called rotary rubber braced damper (RRBD). The RRBD is an economical, lightweight, and easy-to-assemble device. A finite element model considering nonlinearity, large deformation, and material damage is developed to conduct a parametric study on different damper sizes under pushover cyclic loading. The fundamental characteristics of this VED system are clarified by analyzing building structures under cyclic loading. The result show excellent energy absorption and stable hysteresis loops in all specimens. Additionally, by using a sinusoidal shaking table test, the effectiveness of the RRBD to manage the response displacement and acceleration of steel frames is considered. The RRBD functioned at early stages of lateral displacement, indicating that the system is effective for all levels of vibration. Moreover, the proposed damper shows significantly better performance in terms of the column compression force resulting from the brace action compared to chevron bracing (CB).

## Introduction

In the last three decades, structural control systems have been greatly improved to confront natural hazards such as wind and earthquakes [1,2]. Three main types of structural control systems are used: (1) passive control systems, with high energy dissipation density and no need of an external power source; (2) active control systems, with force delivery devices and real-time processing sensors that need power for the actuator to generate a structural control force; and (3) semi-active control systems, that change some structural parameters while consuming less power compared with active control systems. The two general categories of passive damper devices are rate independent and rate dependent. Rate independent type involves metallic yielding or hysteretic and friction devices. The yielding mechanism is such that the energy dissipation component is inactive in confronting low levels of ground motion while in case of high levels of ground motion sliding friction or metallic yielding provides the energy dissipation. This class of damper increases the lateral stiffness of structure. Rise of lateral stiffness along with dissipation of energy may result in lower system deformation, but high story accelerations. Viscous fluid dampers (VFDs) and viscoelastic (VE) solid dampers belong to rate dependent passive dampers. Behavior of VE rubber dampers is a rate dependent phenomenon [3–8]. In recent years, numerical tools such as finite element method have been utilized to optimize the structure of VE rubber damper devices [9,10]. VEDs have made a great contribution in vibration control of aerospace structures [11,12] and civil engineering structures since 1969 [13–17]. VEDs take advantage of shear deformation of viscoelastic materials (VEM). The typical configuration of VEDs is such that the VEM is bonded between steel plates to dissipate oscillation energy. This manner of damping reduces floor acceleration, interstory displacement and interstory shear [18–22]. Advantage of VEDs over VFDs is that application of VEMs results in small increment of structural stiffness and this is due to their inherent storage stiffness, yet to lower degree as metallic yielding devices. In addition, VEDs are able to dissipate energy against all levels of ground motion. The RRBD shares this advantage. Generally speaking, VEDs are known as some of most efficient vibration control devices in strengthening existing and new buildings against wind and earthquake [23]. Recent years have witnessed many practical projects and studies regarding VEDs. These studies have involved development of various configurations [13, 24–28]. Materials of VEDs are readily available and that makes them more common damper devices. However, it is not easy to reach to a balance between stiffness and damping. The RRBD overcomes this issue through sufficient number of parameters to control the balance.

By carrying out a review of bracing systems prior to presenting the proposed system has put the idea into perspective. Braced frames are designed to resist both gravity and lateral loads in beam bending and column compression, and axial compression and tension, respectively. Numerous braced frame types exist including K-bracing, V-bracing, X-bracing, CB and single diagonal [29]. In order to ensure correct bracing type and position, both primary and secondary criterion (lateral drift and architectural objectives, respectively) need to be considered. A comparison study of four bracing types, X-bracing, eccentric bracing, CB and single diagonal bracing, by Alshamrani et al. [30] showed that X-bracing and CB displayed the best performance against lateral force but the former is much more expensive than the others due to the increased number of joints. Additionally, CB displayed greater flexibility for service passages and other openings, like doors and windows, whilst X-bracing was more obstructive. This led the authors to conclude that CB was more efficient, had lower costs and improved architectural aspects compared to the other bracing options.

The nonlinear cyclic response of the brace dictates the dissipation of seismic energy in a Chevron braced frame (CBF). Cyclic axial force deformation behavior of a brace is asymmetrical in tension and compression and buckling effects often result in significant strength and stiffness deterioration [31]. Therefore, when a CBF is subjected to large ground motions, there is a loss of lateral stiffness and a weakening of the frame due to the inelastic buckling of the braces [32]. The early buckling of the braces at specific floor levels [33] make it difficult to obtain well distributed ductility demands throughout the height of the CBF causing soft-story formations, substantial damage to the frame members and dynamic instability [34]. A large number of research studies have been carried out in recent years that have considered new structural configurations [32,35] and hysteretic, friction and VFD passive energy dissipation devices [36–38] and their ability to improve the performance of CBFs.

There is a concurrent determination of structural members, such as beams, braces and columns, during the design of bracing system dampers. This allows the frame to be designed with prior knowledge of the high axial forces generated by the braces. Conversely, a braced seismic protection design should consider how the brace attachment to the initial frames can be affected. When CB systems are used to protect frames from seismic activity, column failure can be induced following an increase in compression forces in the adjacent column to the added bracing member. In addition, the vertical unbalanced force (VUF) can result from CBF brace buckling, with these VUFs having high buckling potential within stories [39]. These situations can be managed by increasing the cross section of the column member, however, this will increase the cost of the build.

This article investigates a VED system for seismic retrofitting of steel moment frame structures using ABAQUS for FE modeling. Static and cyclic tension tests are conducted because they are essential for input into ABAQUS to define the hyperelastic function, in addition, since the likely deformation state for the damper will be simple shear, the finite element analysis (FEA) results of the VEM under shear force are compared with experimental results to ensure the validity of the modeling of the VEM behavior. In columns, compared with chevron bracing, lesser increase in compression force is observed under pushover cyclic loading. Cyclic loading tests of the steel moment frames disclose the fundamental characteristics of the proposed damper. In order to estimate the effectiveness of RRBD under sinusoidal excitation, a small scale laboratory model underwent shaking table tests both with and without the proposed damper system.

## Proposed VE damper system

**Fig 1:**
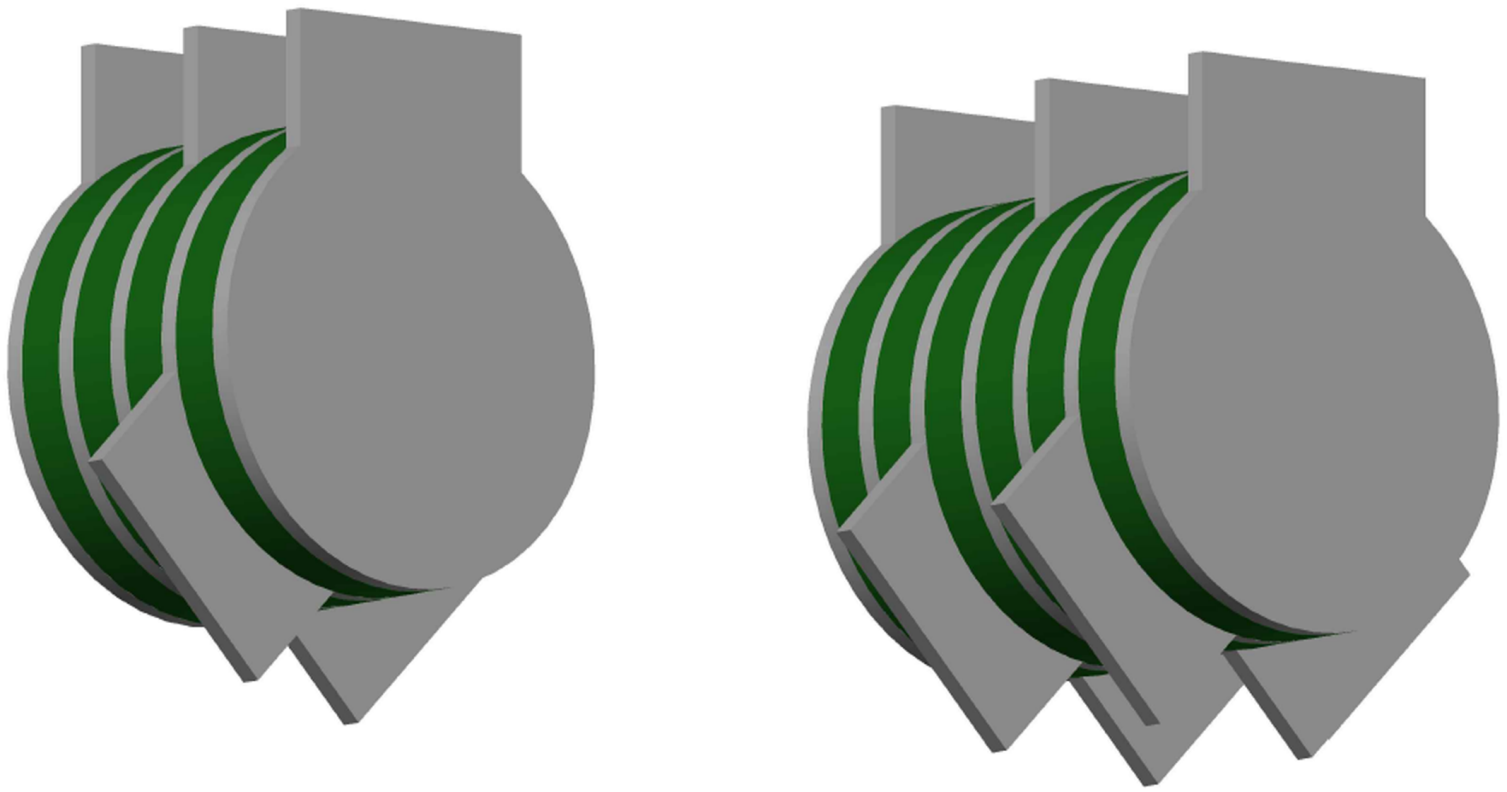
Rotary rubber brace damper (RRBD). Device with (a) 4 VE layers and (b) 6 VE layers.

The suggested damper uses VE pads sandwiched between steel plates to provide shear resistance (Fig 1), ensuring both stable shear force and reduction of lateral displacement and acceleration. These rubber pads produce the restoring force required to return the system to its original location.

**Fig 2:**
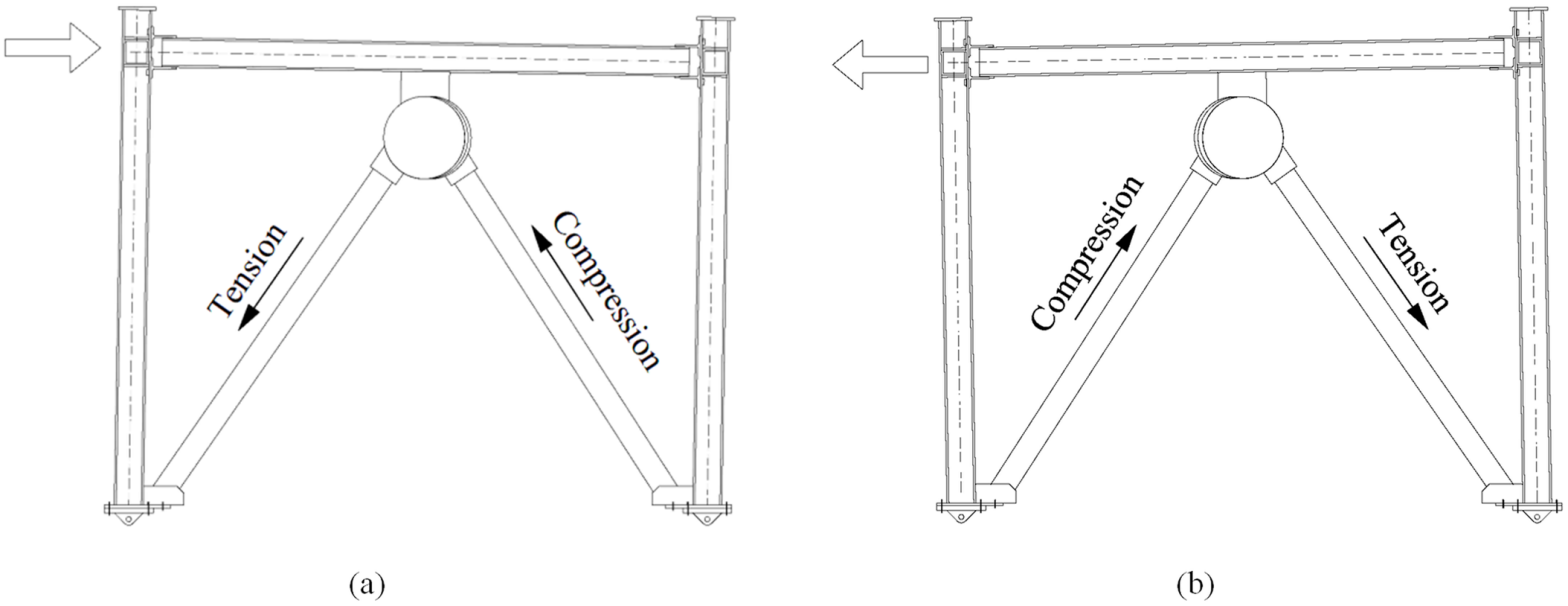
Mechanism of action of the RRBD. Frame movement to the (a) right and (b) left.

The damper is based on the pushing and pulling of chevron brace members as well as slight rotational shear. When a frame structure is displaced in the horizontal direction, the damper steel plates connected to the CBs slide along with the bracing members, allowing the bracings to pull and push the rubbers; thus, energy dissipation occurs as bracings are subjected to tension and compression. Fig 2 shows the performance mechanism of this system.

The following sections will show that the VEM has a moderate initial stiffness at strains lower that 60%. However, the stiffness is reduced at mid-range strains around 90% and strains in excess of 90% cause the rubber to have a stiffening or hyperelastic effect. Nevertheless, the specific VEM compound largely dictates the levels at which these stiffness changes occur. As such, the VEM stiffness behaviors have been incorporated into the RRBD design, which will be discussed further in the next sections.

The following parameters are used to control the behavior of the RRBD system:

- Rubber thickness (T)
- Rubber diameter (D)
- Aspect ratio (D/T) of the device
- Number of rubber pads that directly affect the shear area
- Strength of VE material; cored holes in the VEM can reduce the stiffness (k)
- Different types of rubber, e.g., natural rubber, high-damping rubber
- Dynamic constitutive behavior of VEM

## FE modeling

ABAQUS ver. 6.12 is used to conduct FE modeling of the materials used in the construction of the damper [40]. The cyclic displacement control loading is imposed to generate cyclic behavior of the moment resisting steel frame. An incremental increasing the displacement to achieve the maximum deformation capacity of the model is used. For this purpose, the lateral displacement was applied at the both column tips, in addition, pin supports were assumed at both column ends. Surface-based tie constraints were applied between all contacted regions, i.e. the beam-to-column T-stub connections, the base-plate column connections, the connections between bracing members and gusset plates, and the gusset plates connected to the column flanges. On the whole, surface-to-surface discretization provides more accurate stress and pressure results than node-to-surface discretization if the surface geometry is reasonably well represented by the contact surfaces [40]. This requires the definition of a master and a slave contact surface. The choice of the master and slave surface is made considering the mesh discretization and stiffness, i.e. the surface with the higher stiffness or coarser mesh was chosen as master surface because it results in a smoother solution. It should be noted that the solution could become quite expensive if the slave surface was much coarser than the master surface. Moreover, the stiffness of the structure and not just the material should be considered when choosing the master and slave surface. These were accounted for in the model. The influence of temperature was not considered in the characterization process assuming that the proposed control system would be always installed inside buildings.

### Modeling of VEM

This section discusses the development of an analytical FE model for the RRBD. It is necessary to understand the behavior of rubber in order to create a device that uses rubber materials; therefore, the material properties of the rubber need to be ascertained through the use of appropriate models in order to analytically model a VEM.

A suitable numerical rubber model should consist of hyperelasticity that describes the variation of the elastic modulus at large strains and the viscosity of the rubber. Hybrid elements are used for nearly incompressible materials, like rubber with high Poisson ratio. Simplistically, under loading conditions, rubber exhibits a high volume change resistance with this assumption of incompressibility being considered an important aspect of rubber’s mathematical models. Many different models such as Arrude-Boyce, Van der Waals, Mooney-Rivlin, Neo-Hookean, Ogden, polynomial, reduced polynomial, and Yeoh exist for the hyperelasticity of rubber [40].

For numerical simulation, FEA is performed to obtain the shear stress—strain relationship for various elongation levels of the specimen and to evaluate shear behavior of VEM, because the deformation state for the device will be shear. The FE model considers both material and geometric nonlinearities. However, owing to the incompressibility of rubber, i.e., Poisson’s ratio close to 0.5, conventional solid elements can result in large errors. There is some uncertainty regarding the actual value of rubber's Poisson's ratio [41]. In this study, a Poisson ratio of 0.495 was assumed for rubber material. Using linear elements with reduced integration points and coarse FE meshes for problems associated with large deformations, such as the one in this study, is not recommended as this might lead to hourglassing error by providing no stiffness against certain deformation modes. To determine the suitable type and dimensions for the rubber layer, a sensitivity analysis was conducted, and the 3D-stress20 node element (C3D20RH) was selected. This element supports plasticity, hyperelasticity, creep, stress stiffening, large deflection, and large strain capabilities with mesh size of 5 mm. Therefore, the nonlinear behavior of VEM can be modeled. To show the reduced polynomial model material constants, the average values of the whole specimen samples were calculated, and these were later used in the FEA of the RRBD system. Yeoh performed a series of tests on vulcanized synthetic rubber and proposed the following cubic function that is a function of *I*_1_ in the from of (*I*_1_-3) and material constants C_10_, C_20_, and C_30_:

$$W=\;C_{10}\left(I_{1}-3\right)+\;C_{20}{\left(I_{1}-3\right)}^{2}+C_{30}{\left(I_{1}-3\right)}^{3}$$ (1)

where I_1_ is the first invariant of the right Cauchy-Green deformation tensor, and C_10_, C_20_, and C_30_ are material constants that are determined from experimental data when a rubber specimen is under uniaxial load. Furthermore, in contrast to other higher-order models, Yeoh’s model is only dependent upon the first strain invariant and can fit many different deformation models by using only uniaxial tension test data. This reduces the material testing requirements. For uniaxial tension, Eq (1) can be expressed as [42,43]:

$$\sigma =2\left(\lambda^{2}-\lambda^{-1}\right)\;[C_{10}+2C_{20}\left(I_{1}-3\right)+3C_{30}\left(I_{1}-3{)}^{2}\right]$$ (2)

where λ is the stretch ratio, which is defined as the ratio of the current to the original length, and I_1_ is a function of λ and is expressed as I_1_ = λ^2^ + 2λ^-1^.

### Modeling of steel material

To perform a 3D simulation of the steel parts of the device, an eight-node first-order 3D linear hexahedral element with three degrees of freedom per node is used. This is the cubic element C3D8R, with one integration point in the middle of the element in addition to those at the corners. This element was chosen instead of C3D20R because beam-column corners are slightly bent and because the analysis has lower time cost despite providing a sufficiently accurate result.

## Experimental setup

### Uniaxial specimen testing

**Fig 3:**
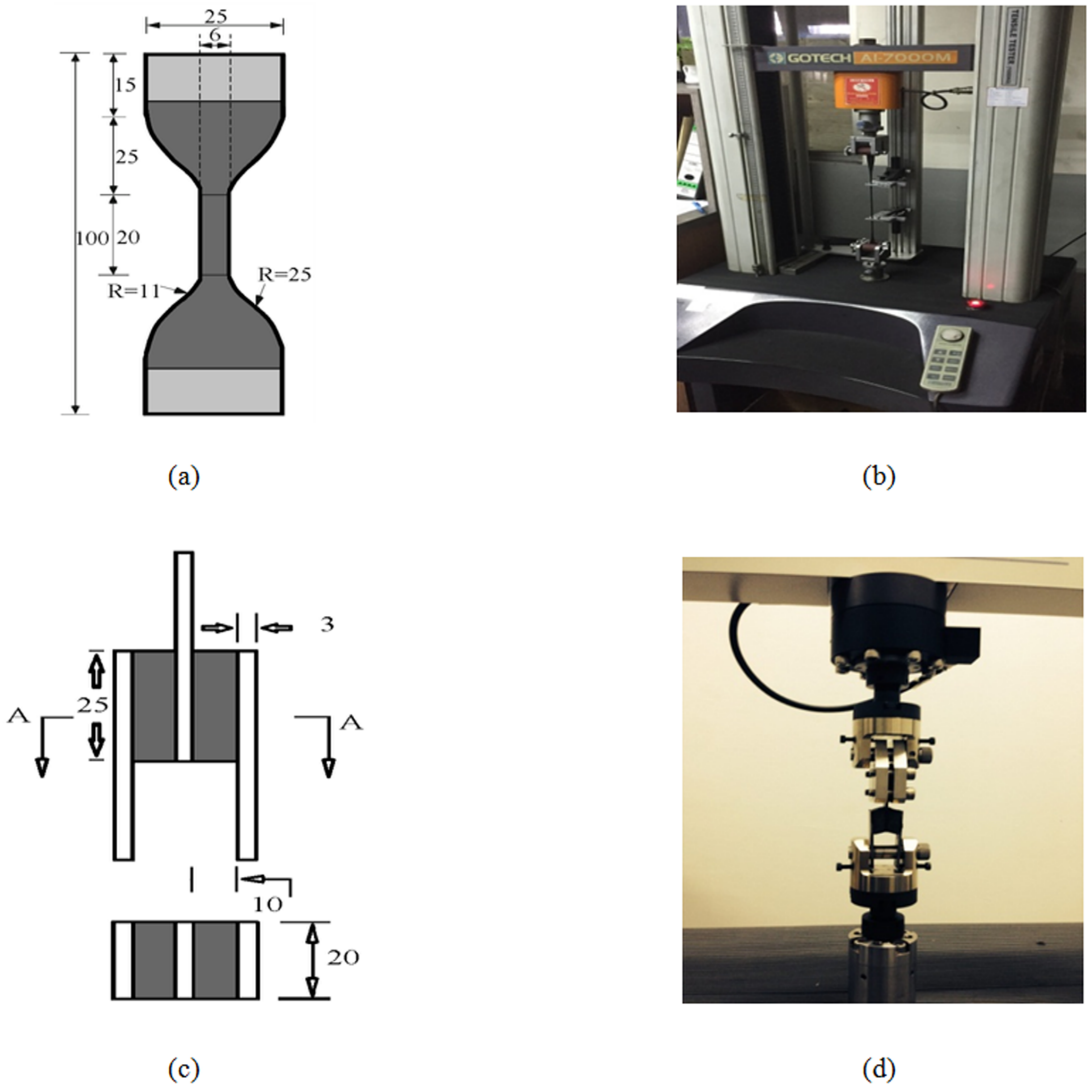
(a) Specimen sample for uniaxial tension test (mm); (b) photograph of dumbbell specimen sample; (c) Simple Shear Specimen Dimensions; and (d) photograph of specimen sample.

To obtain the material properties of the VEM, both monotonic and cyclic uniaxial tensile tests were conducted based on the procedure detailed in ISO37 [44], and the stress-strain response for each rubber specimen was measured using a universal tensile testing machine, shown in Fig 3(b). During testing, the environmental temperature was fixed at 25°C. After measuring the sample thickness, the specimen was placed between the tensile grips. The strain was measured using an extensometer with clip gauges initially separated by 20 mm. There was a real time measurement of the distance between the gauge marks up to the breaking point. The monotonic uniaxial tension test was applied until all three specimen samples reached the failure state. For preparing uniform 2-mm-thick dumbbell specimen samples for uniaxial tension tests, synthetic rubbers were used according to the Malaysia Industrial standards, as shown in Fig 3(a). The shaded area indicates the area clamped by the jig. In the tensile test, the gauge length (I_exp_) and tensile force (F_exp_) were measured.

From the measured I_exp_ and F_exp_, the nominal stress P_exp_ and stretch λ_*exp*_ were calculated using Eq (3):

$$P_{exp}=\;\frac{F_{exp}}{t.B}\;,\;\lambda_{exp}=\frac{I_{exp}}{I_{0}}$$ (3)

where B is the thickness of the specimen and I_0_, the initial gauge length.

**Fig 4:**
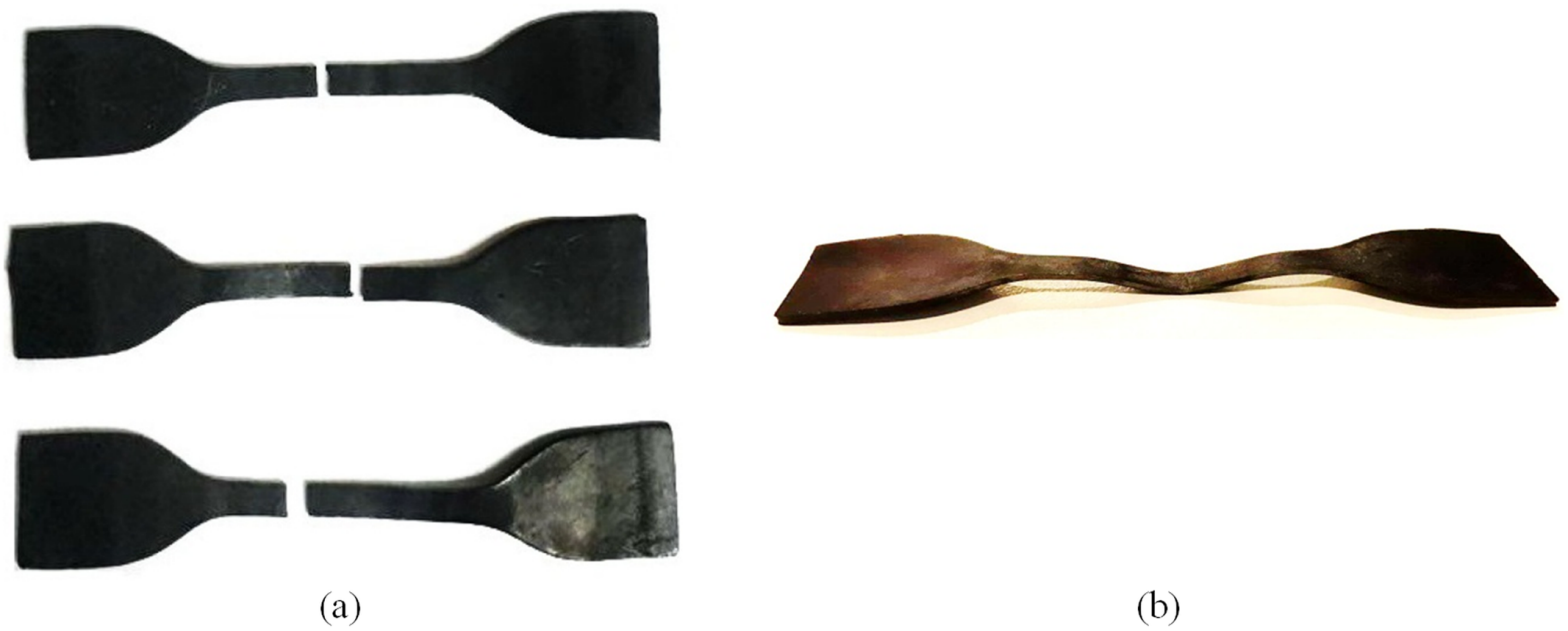
Rubber specimens tested. (a) Monotonic; (b) cyclic.

**Fig 5:**
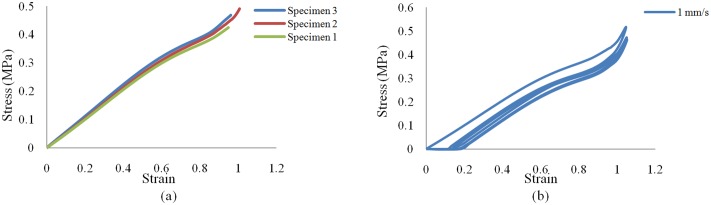
Uniaxial experimental stress—strain curve results. (a) Monotonic; (b) cyclic.

All four rubber specimens collected for the uniaxial test had the same thickness with maximum difference of 0.2 mm. The specimens tested are shown in Fig 4. A cyclic tension was applied on dog bone specimen. The steady rate of elongation at 1mm/s was carried on for all the tests. It should be noted that the unknown strain field around the grips, the compliance that may exist in the loading cables, and the material flowing from the grips cause uncertainty in evaluating the relationship between the actual strain in the center area of the specimen and the grip travel. The stress-elongation relationship for the four samples is demonstrated in the tensile test results given in Fig 5.

ABAQUS provides a convenient ‘Evaluate’ option that allows the users to view the behavior predicted by a hyperelastic or viscoelastic material and that allows researchers to choose a suitable material formulation. It is possible to use the Evaluate option to calculate the material’s response based on the experimental data using the strain energy potential that researchers have specified in the material definition. Any strain energy potential coefficients and any material instabilities identified during the tests should also be reported. Table 1 shows the specified constants for a single data set gathered by evaluating the experimental data.

**Table 1:** Reduced polynomial model (Yeoh) material constants.

Rubber material properties	C_10_	0.3524
	C_20_	-0.02091
	C_30_	0.0012
	D_1_	0.0015
	D_2_	0
	D_3_	0

### Shear specimen testing

A standard double sandwich specimen was used as the shear specimen. Fig 3(c) shows the dimensions. The sandwich construction consisted three steel plates and two rectangular specimens fixed to the base of testing machine. In order to apply the shear deformation to the specimen, the middle steel plate had to be stretched by the movable clamp of the machine. The thickness of the rubber pad was 10 mm where the shear area for each layer was 500 mm^2^. During the vulcanization process, the rubber was bonded to the steel plate and the bond was stronger than the rubber. A number of well-known earthquake events, such as the Northridge (USA, 1994) and the Kobe (Japan, 1995), lay within a frequency range of 0.14–1.12 Hz. As such, the rate dependent behavior of the sample was evaluated at five test rates (0.1, 0.2, 0.5, 1.0 and 1.5 Hz) and at various strain levels (25%, 50% and 100%) as illustrated in the shear test set-up in Fig 3(d).

### Shear stress relaxation test on the VEM

**Fig 6:**
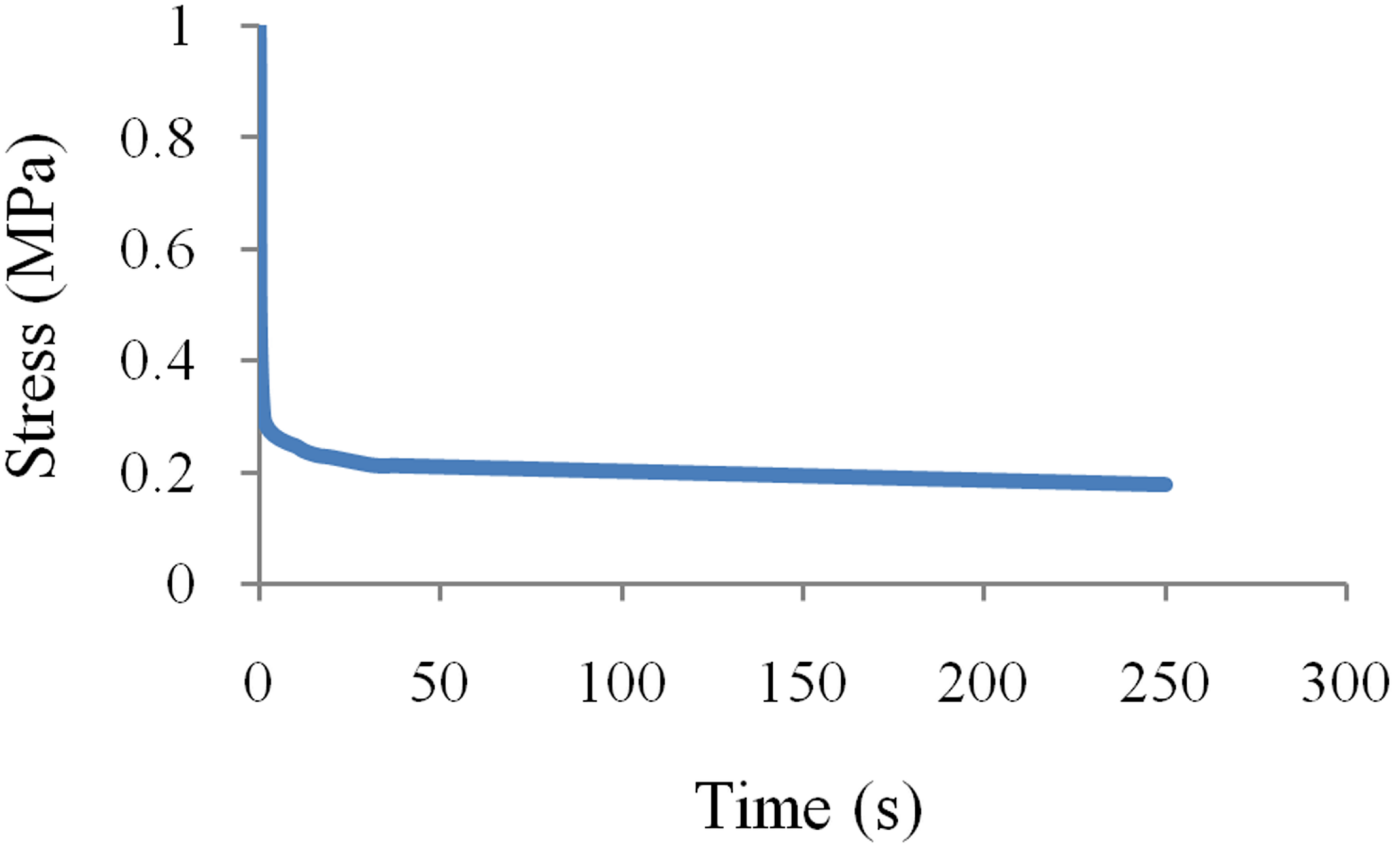
Relaxation shear test result.

The material's nonlinear elasticity is represented by hyperelastic material model but no time dependence. The overall material behavior in terms of nonlinear elastic and strain-rate dependencies can be predicted by the hyperelastic plus VE material model. In other words, a single stress relaxation curve can present the VE properties of the rubber regardless of the strain level. The time dependent stress response is measured in a stress relaxation test under constant strain level of specimen. To do so, a mechanical strain is suddenly imposed to the middle steel plate and held constant. Stress relaxation test was conducted at 30% strain for 250 records. The results of the test are depicted in Fig 6.

## Results and discussion

**Fig 7:**
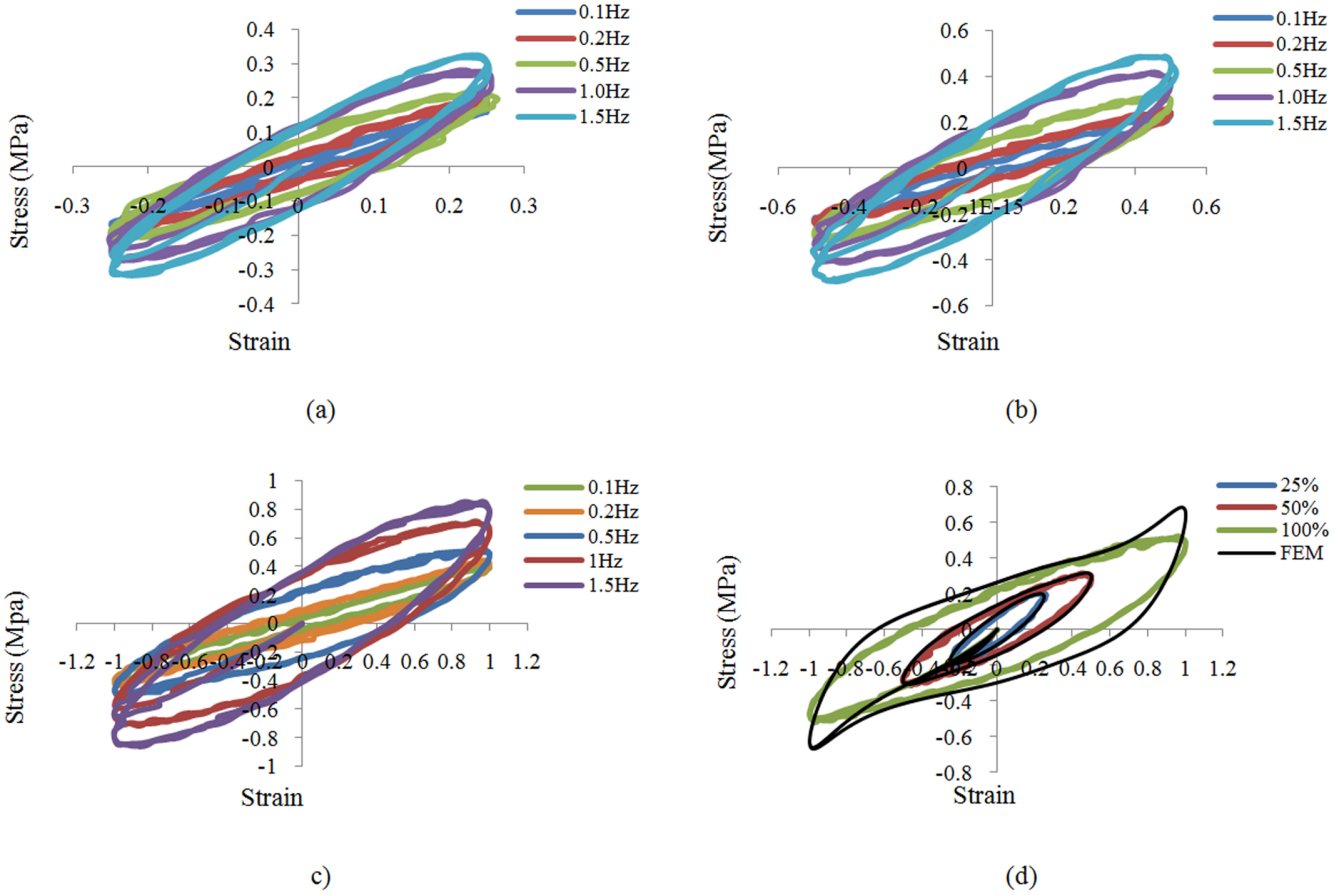
Experimental shear test results at various rates. (a) 25% strain, (b) 50% strain, (c) 100% strain; and (d) comparison of shear test results and FEM at 0.5Hz.

The results are discussed in four parts: verification of VEM modeling, verification of moment resisting steel bare frame (BF), cyclic analysis of frame equipped with a damper and the shaking table test. The hyperelastic function in ABAQUS is determined by only tension inputs. However, accurate modeling of the shear behavior is critical due to the probable damper state which is simple shear. Fig 7(a)–7(c) show shear strain-stress curves at various rates and cyclic strains of ± 25%, ± 50% and ± 100%. It is considered that the chosen VEM has a fine energy dissipation capacity as the hysteresis curves display full ellipse. In addition, the different excitation rates and amplitudes cause differing degrees of slope and pump curve, indicating that excitation rate and amplitude affect stiffness and damping change. An increase in the excitation rate causes an increase in the enveloping area and tilt angle of the hysteresis curve, as shown in Fig 7(a)–7(c). This suggests that the excitation rate is correlated with energy dissipation capacity and stiffness.

In order to evaluate the occurrence of stress softening in both directions, the samples were tested to both extremes and results revealed that strain in negative direction had a softening effect in the positive direction. Any increase in displacement amplitude causes a decrease in the tilt angle and an increase in the enveloping area of the hysteresis curve as indicated by Fig 7(d), which displays the comparison of hysteresis loops between the simulation and test at 0.5 Hz. The fits at all the strain levels were reasonable, but the fit for the hysteresis loop is best at strain level of 25% and 50%. The 100% displacement level behavior deviates the most from the test data. It can be seen from the Fig 7(d) that the level of correlation needed for analysis will not be achieved at the 100% displacement level. This will be accounted for in the device design in such a way that even at maximum frame displacement the damper will never reach this level of strain. As seen in Fig 7(d), reasonable agreement is found between the experimental shear test and FEM results, with the biggest discrepancy being the dynamic stiffness. This can be explained by the rough linear fit of the hyperelastic model stiffness.

### BF verification

**Fig 8:**
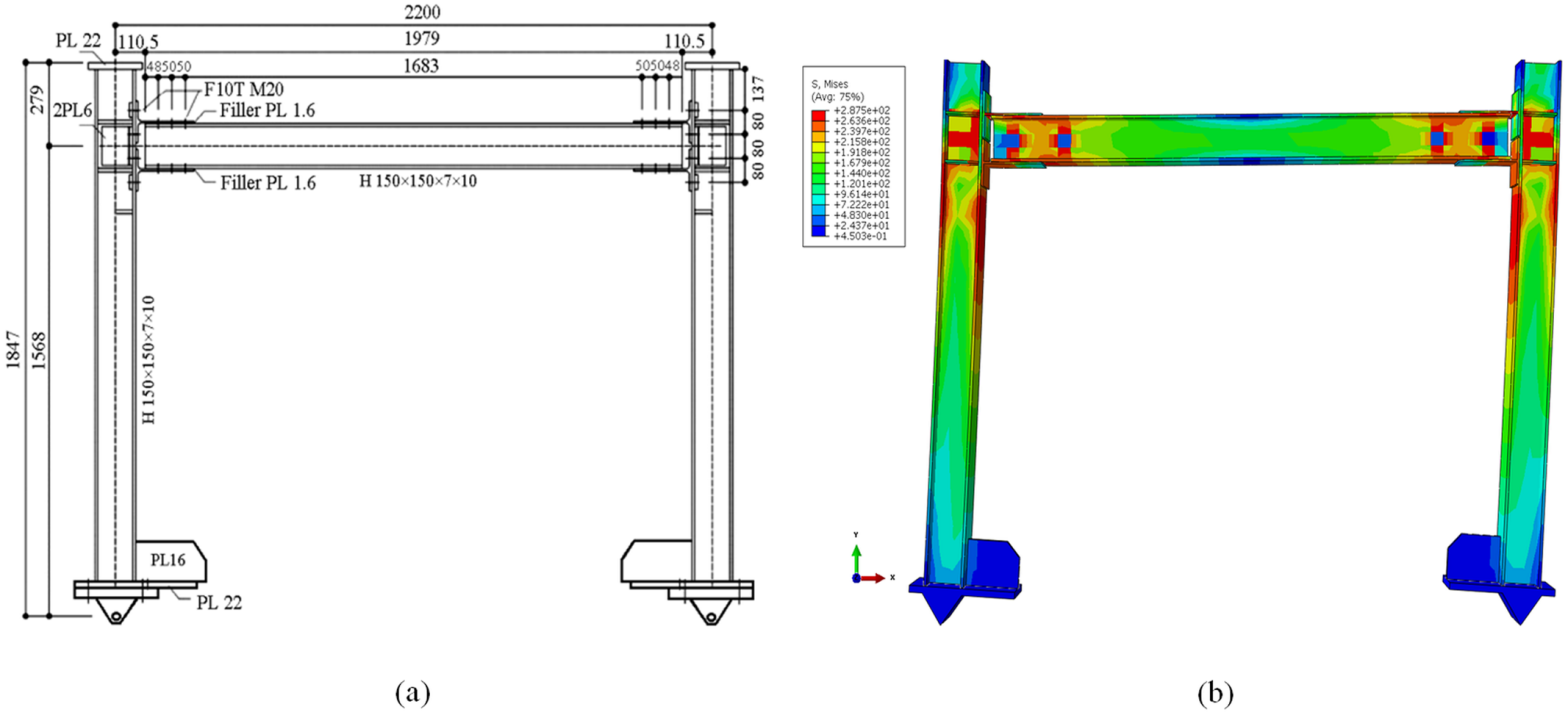
Steel moment BF. (a) Details of frame tested conducted by Hou and Tagawa (2008) [45], and (b) FE created by authors (all dimensions are in millimeters).

The geometrical properties of the FE model in this study are similar to those of the steel moment frame tested by Hou and Tagawa. Bolted T-stubs cut from H-300x150x6.5x9 (steel grade: SS400) connected the column and beam members of H-150x150x7x10 (steel grade: SN400B) [45]. Fig 8 shows the details and dimensions of authors FE model and Hou and Tagawa’s steel moment frame test.

**Fig 9:**
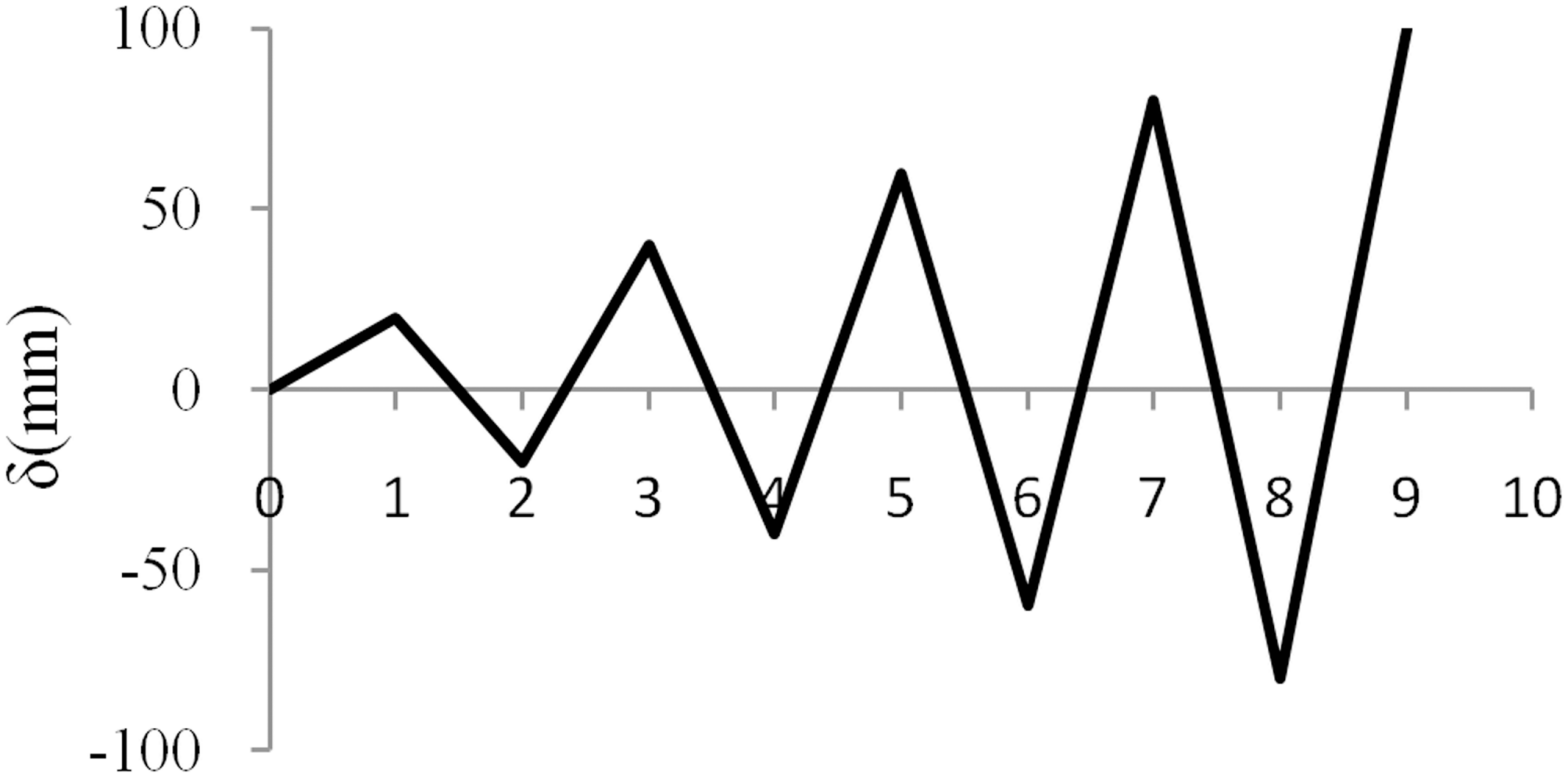
Cyclic loading protocol [45].

Pushover cyclic loading and a parametric study for a frame with a damper are conducted, and the results are compared. The latter includes different components of the RRBD, and it investigates the effects of the rubber thickness and rubber diameter of the RRBD on the frame behavior. The models are subjected to cyclic loading with the same protocol as in Hou and Tagawa’s experimental test [45], as shown in Fig 9.

Table 2 shows Young’s modulus, Poisson’s ratio, and the density for steel and rubber.

**Table 2:** Mechanical properties of components of RRBD.

Material	Young’s modulus (MPa)	Poisson’s ratio	Density (g/mm^3^)
Low-carbon steel	210,000	0.3	7.85
VEM	1.25	0.495	0.001

**Fig 10:**
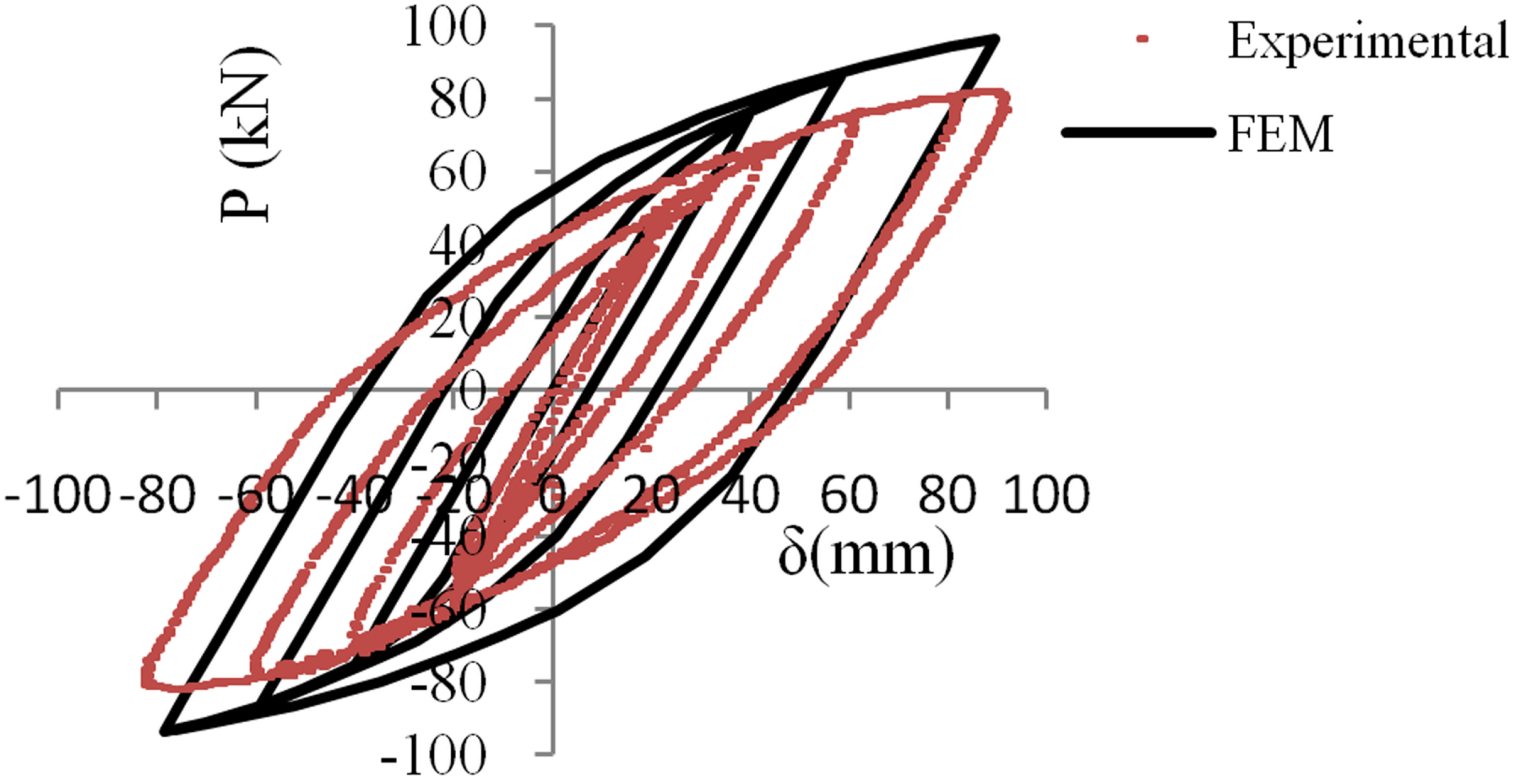
Comparison of experimental and numerical results.

Fig 10 shows a comparison between the experimental and the numerical results. The results show good agreement.

### Parametric study of rubber thickness and rubber diameter under cyclic loading

**Fig 11:**
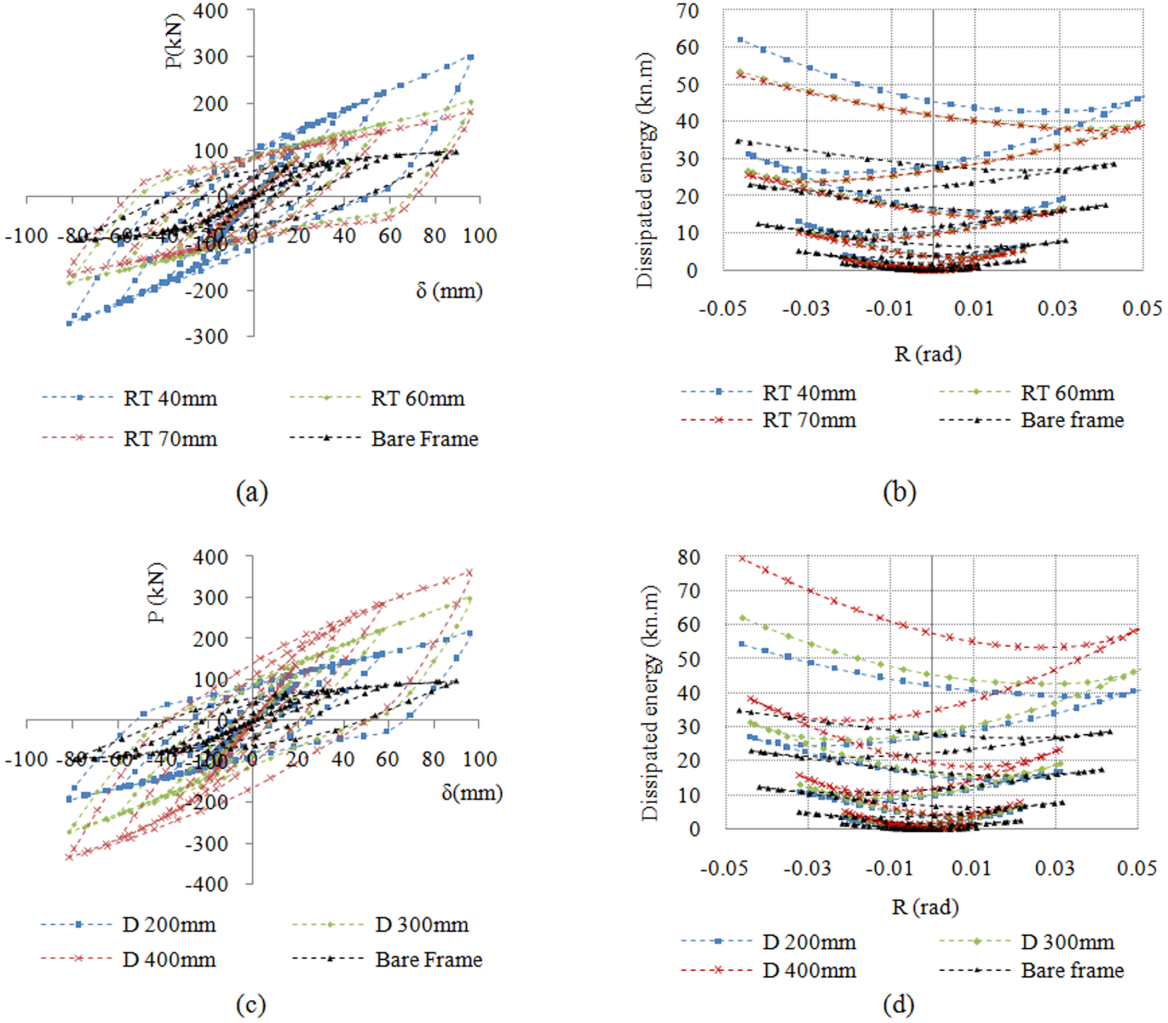
Cyclic behavior of BF and frame with damper. (a) effects of rubber thickness (RT); (b) dissipated energy vs. rotation; (c) effects of diameter of rubber pad (D); and (d) dissipated energy vs. rotation.

This section discusses the numerical analysis results for the considered moment frames subjected to cyclic loading. A comparison of the results indicated to reveals the characteristics and advantages of the damper system. In theory, the dimension and number of VE layers is not limited, which suggests that by utilizing numerous rubber pads of varying dimensions it would be possible to design an RRBD with different stiffness and energy dissipation values. In order to prevent any buckling of the brace, it is necessary to ensure that the buckling stiffness of the brace is higher than the total stiffness of the whole damper. Therefore the frame’s stability will be increased by evaluating the proper behavior of the brace. This also was accounted for in the design of the device by identifying that the buckling point of the brace was never reached even at maximum frame displacement. In Fig 11, the left panels show the lateral load-displacement hysteresis diagram for all frames under cyclic loading, and the right panels show the corresponding energy dissipation and story rotation angle relationship. All dampers functioned at relatively small displacements and then showed stable hysteresis loops for relatively large displacements. Fig 11(a) shows the cyclic load-displacement relationship for different rubber pad thicknesses; it clearly shows that with decreasing VEM thickness, the energy dissipated by the system increases substantially. As shown in Fig 11(a), the force values of the hysteresis curve for the frame with RRBD with a rubber pad having 40-mm thickness and 300-mm diameter is approximately 3 times that of a BF without a damper at the maximum displacement. The area of the loop associated with the damper-equipped frame increases along the simulation duration, showing the high energy dissipation capability of the damping system. Fig 11(c) shows the effects of the diameters of the VEM pads. As the diameter was increased, more energy was dissipated because of the larger shear area, and the corresponding force required for the same amount of frame displacement increased notably, indicating higher stiffness. More importantly, the RRBD exhibits larger stiffness and strength with greater imposed deformation. This phenomenon corresponds to the performance nature of VEMs, which demonstrate increasing stiffness magnitudes at higher levels of strain. This is a unique property that, to the best of the authors’ knowledge, other ordinary dampers do not possess. For example, frictional dampers do not exhibit any stiffness upon sliding. In most metallic dampers, stiffness either decreases or remains the same at larger displacements. In the case of VEMs, however, higher reliability in structures subjected to severe earthquakes is ensured by increasing stiffness. The RRBD prevents extra-large displacements and collapse of a structure by amplifying the structural stiffness at large displacements.

Table 3 lists the FEA results with regard to the geometrical and mechanical properties of frames obtained from the numerical study. The results are given in terms of the initial stiffness (k), maximum load (P_max_), energy dissipation (E_D_), and ratios of each value with respect to the BF. The stiffness can be measured as the average slope of the displacement-force hysteresis loop, while the energy dissipated per cycle of motion is the area of displacement-force hysteresis loop that is defined as:

$$E_{D}=\int f_{d}dx$$ (4)

**Table 3:** Mechanical properties of parametric study models.

Model no.	D (mm)	t (mm)	D/t	k (kN/mm)	k/k_A_	P_max_ (kN)	P_max_/$P_{max}^{BF}$	E_D_ (kJ)	E_D_/E_BF_
BF	N/A	N/A	N/A	1.05	1	98	1	8.5	1
1	300	40	7.5	3.1	2.9	300	3	14	1.6
2	300	60	5	2.1	2	200	2	24	2.8
3	300	70	4.3	1.85	1.7	180	1.8	23.2	2.7
4	400	40	10	3.75	3.5	360	3.6	32	3.7
5	200	40	5	2.2	2.1	213	2.2	26.6	3.1

N/A: not available

Any subsequent variation in the VEM volume along with an increase in the thickness and diameter affects parameters such as the stiffness, maximum load, and energy dissipation of the damper. An increase in the VEM thickness, as opposed to an increase in the diameter, can result in a reduction in the maximum induced load and stiffness.

### Shaking table test

**Fig 12:**
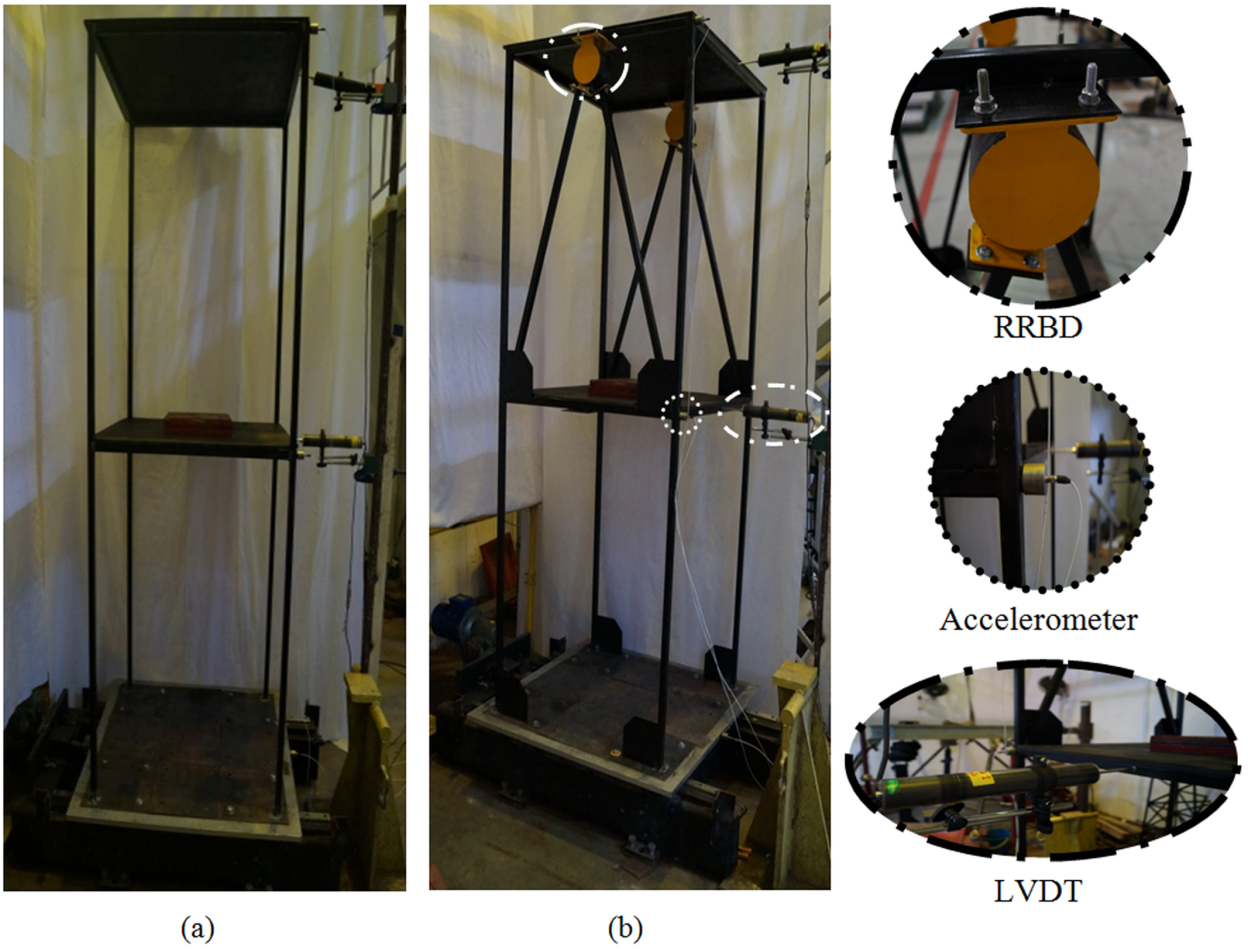
Shaking table test frames. (a) BF, (b) frame with damper.

Fig 12 shows a number of shaking table tests, which simulated sinusoidal excitations, were carried out to identify the efficacy and feasibility of the proposed damper in earthquake situations. Two storey steel frames with different configurations, including BF and frame with damper, were used as the model structures for the shaking table test. The BF was used as a control to compare the results of the RRBD embedded frame structure. Each storey height was 1.2 meters and three accelerometers were used in the test to measure the model structure’s acceleration responses in the direction of input ground motion. The floor deflection was also measured during the tests using three linear variable displacement transformers (LVDTs) with a dynamic range of ±30 cm. A five second harmonic displacement was applied to the frames, which were then left for free vibration.

**Fig 13:**
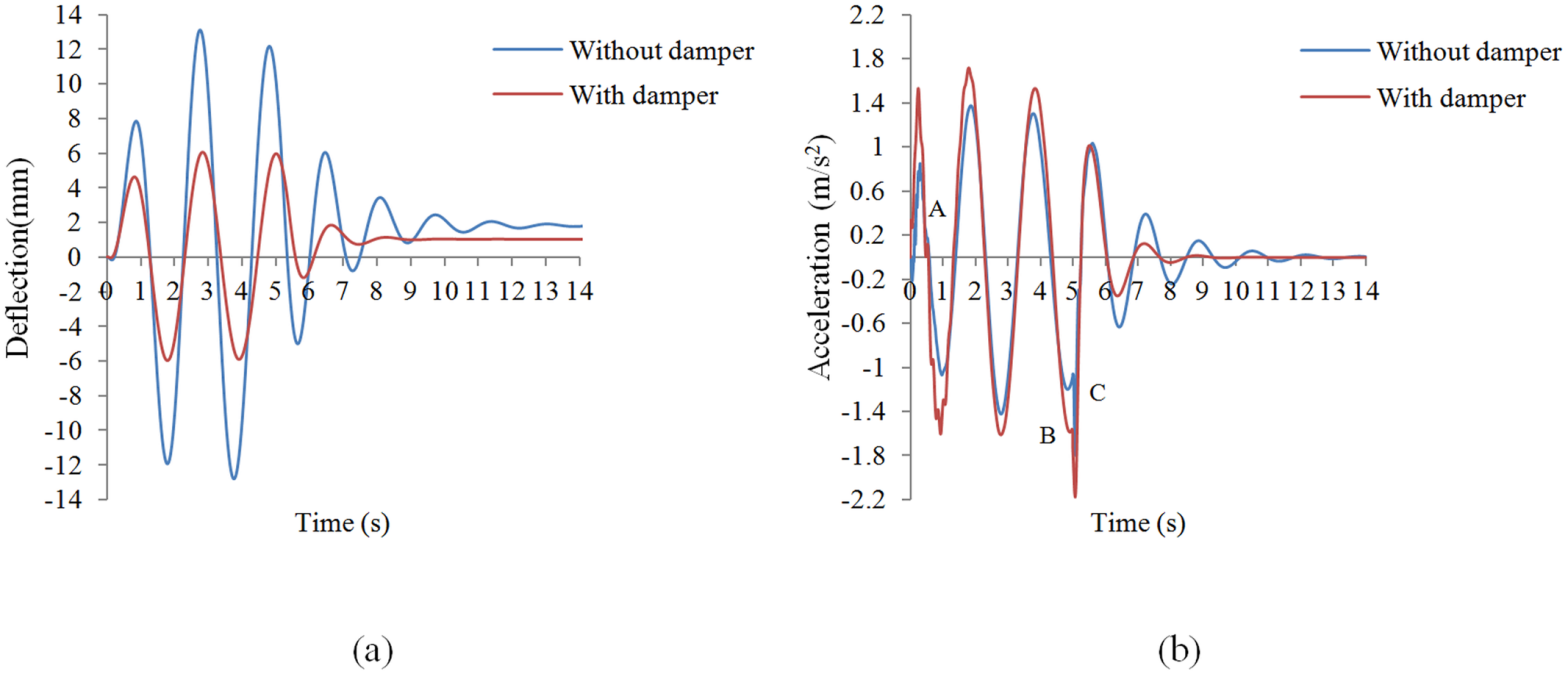
Comparison between the BF and frame with damper under sinusoidal excitation. (a) Deflection, (b) acceleration.

Fig 13 shows the tip deflection and acceleration results following sinusoidal excitations. The results showed that when the RRBDs are installed on the frame there is effective reduction of the displacement response of the top floor (Fig 13(a)). Indeed, the top floor displacement response reduces by 53.89% (13.12mm to 6.05 mm). The test also showed that the RRBD had an effective re-centering effect on the vibration centre and is able to prevent any residual deformation. The acceleration responses show a slight increase in Fig 13(b) with the maximum top floor acceleration increasing from 1.38 m/s^2^ to 1.71 m/s^2^. This was due to an increase in stiffness and damping of the structures caused by the RRBD. As can be seen, a disturbance occurred at the beginning of the movement of the shaking table (point A) and at points B and C. After inspection, it was determined that this was due to the sudden movement and sudden stoppage of the shaking table at the start point of the shaking table movement and at time of five seconds, as captured by the sensitive accelerometer. Whilst the increase in stiffness may cause an increase in acceleration responses, it is considered that the increased stiffness and damping of the structures will effectively mitigate displacement responses.

The displacement reduction, initial stiffness level, deformation capacity ratio and other properties of RRBD obtained from the experimental test and simulations are listed in Table 4 and compared to common passive dampers. Overall, it can be said that the RRBD has an excellent performance and the benefit of a low cost.

**Table 4:** Properties of RRBD and major types of passive dampers.

Parameter	Damper Type			
	VED RRBD	Metal damper (Slit) [46–48]	VFD [49]	Friction damper [50,51]
Displacement reduction	54%	74%	50–70%	50%
Initial stiffness level	Low	High	Vey low	Moderate
Deformation capacity ratio	500%	12%	Stroke restriction	Stroke restriction
Construction cost (US Dollar) (bracing included)	1600 ^a^	2,000	14,000–18,500	6,800
Coverage of vibration level	ALV ^b^	MHV ^c^	ALV	MHV

^a^ Based on Malaysian market;

^b^ ALV: all level of vibration;

^c^ MHV: moderate and high level of vibration

**Fig 14:**
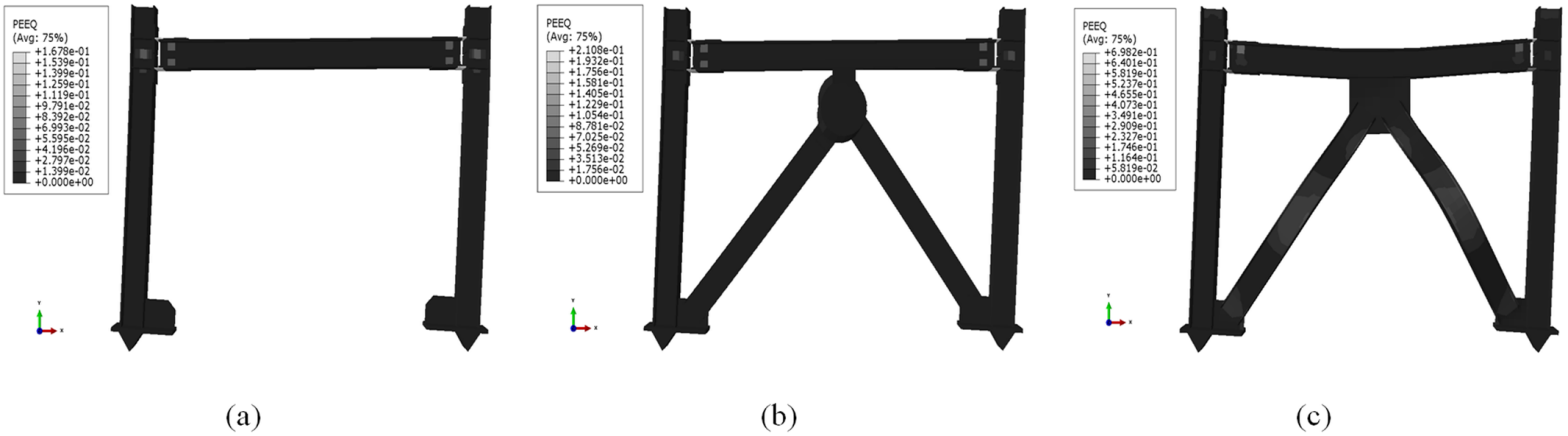
PEEQ distribution at 85-mm column tip displacement. (a) BF; (b) frame with damper; and (c) typical chevron bracing.

Fig 14 shows the deformed shape of the frame models at maximum column tip displacement. Equivalent plastic strain (PEEQ) contours obtained from the simulation are depicted for the peak displacement. The results obviously demonstrate that plastic deformations, or in other words, damage of the frame with added dampers resemble those of the BF; moreover, a significant reduction in damage is observed compared with a frame with typical chevron braces in which both the beam and the braces are completely damaged, hence protecting the integrity of other main structural components.

### Skeleton curve

**Fig 15:**
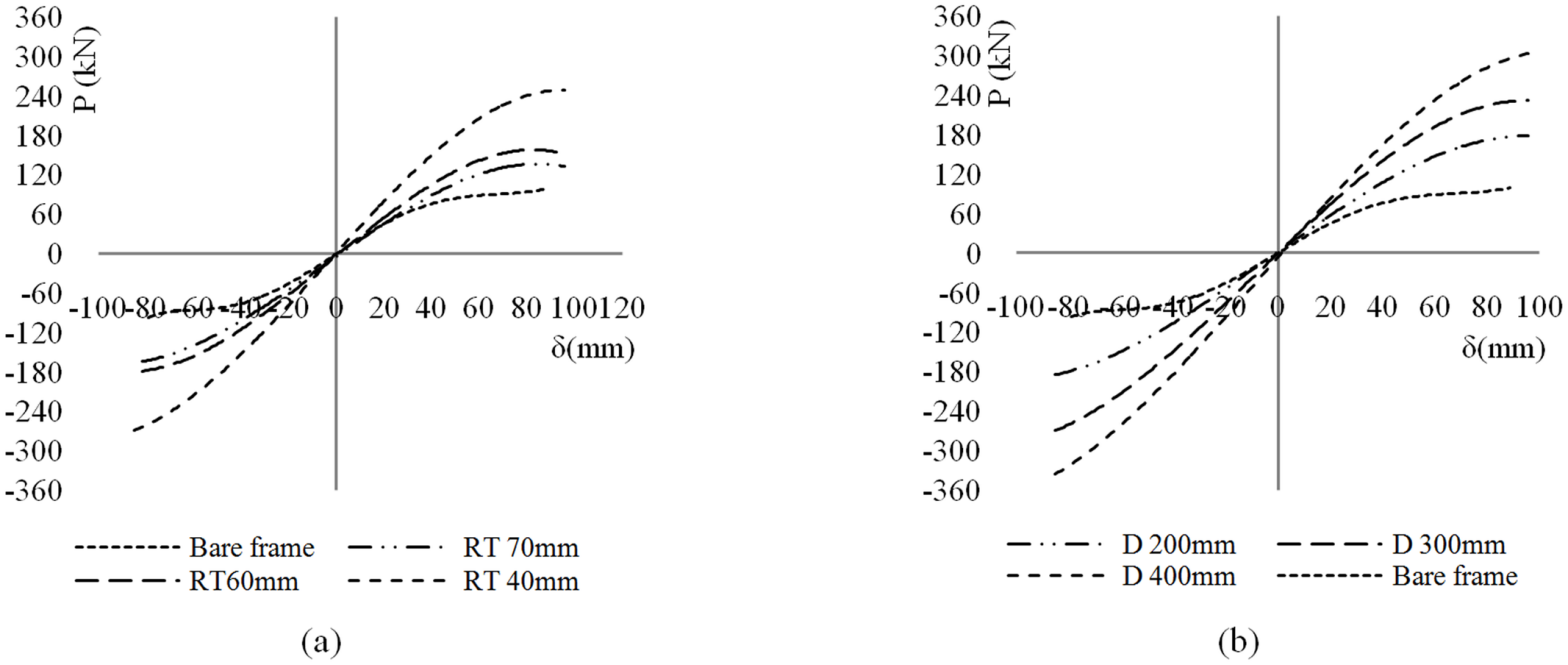
Skeleton curves. (a) different rubber thickness, and (b) different rubber diameter.

Skeleton curves of the seven specimens were obtained by connecting the peak points at each loading increment of the hysteretic curves, as depicted in Fig 15. Fig 15(a) shows a comparison of the backbone curves of the load—deformation behavior for different rubber thicknesses; it shows that the strength decreases significantly owing to an increase in the rubber pad thickness. This is attributable to the rubber stress—strain behavior. Considering that the device works using shear movement, for the same frame displacement, reducing the thickness increases the deformation rate and strain of the rubber. Moreover, reducing the VE layer thickness at a certain point is not recommended, because beyond a certain strain, the VEM will not function linearly any more. Fig 15(b) shows that the load-carrying capacity increases with the rubber pad diameter. This indicates that frames with energy dissipation devices have higher load-carrying capacity and initial stiffness than a BF owing to the additional stiffness of the RRBD. Furthermore, the skeleton curves of damper-equipped frames confirm the functionality of the dampers at very low displacements.

### Compression force of left-side columns

**Fig 16:**
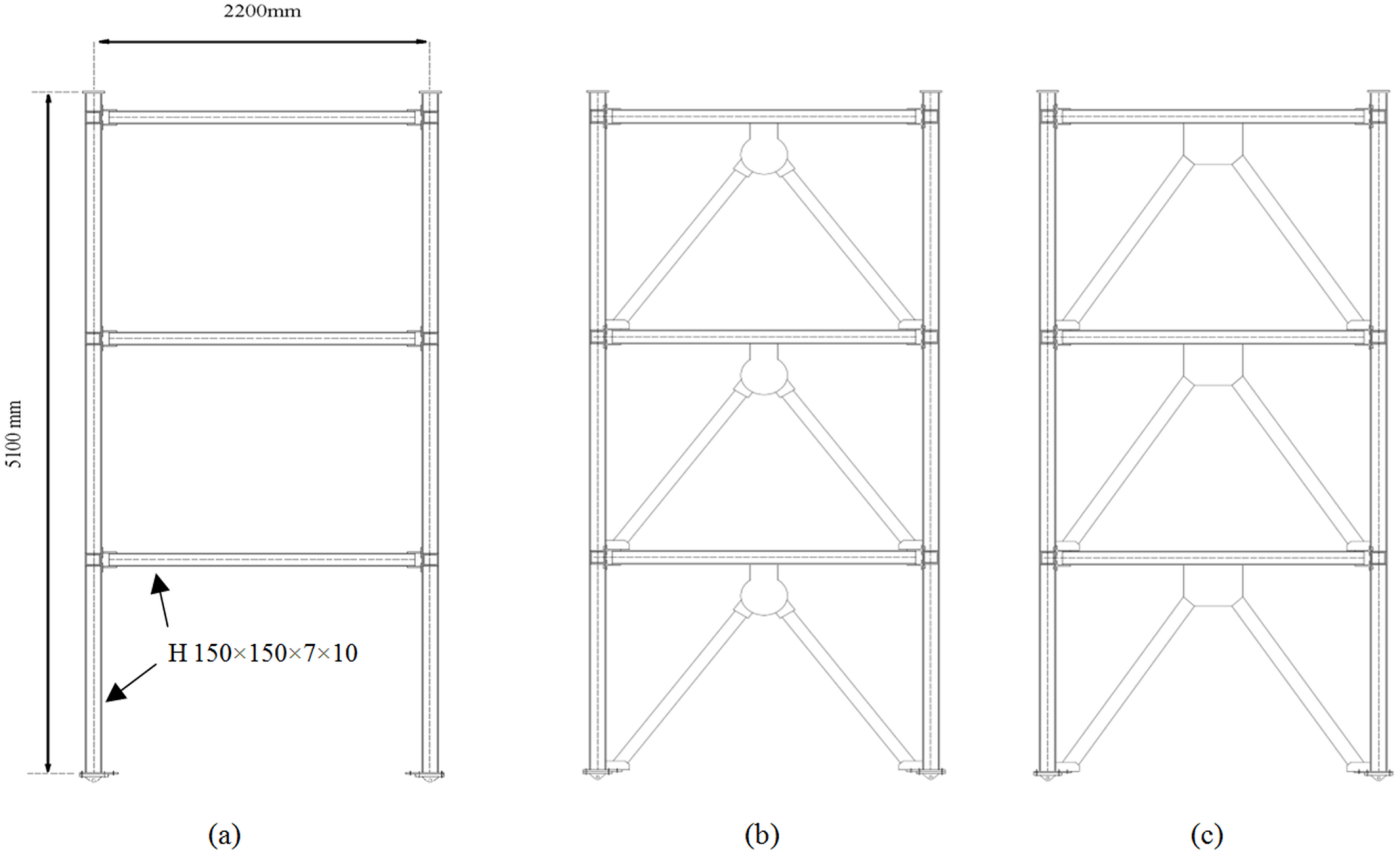
Frame models for cyclic analysis. (a) BF, (b) frame with damper, and (c) typical chevron-braced frame.

Cyclic analyses were conducted for the considered three-story model frames, as depicted in Fig 16. The steel moment frames were used to highlight the performance improvement associated with adding a damper. The following models were analyzed: BF, frame with added damper, and typical chevron-braced frame. Same cross section was considered for all column elements from the first to the third story.

**Fig 17:**
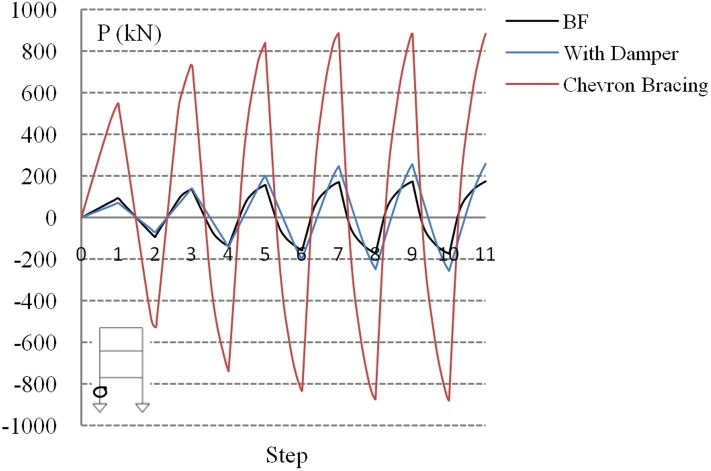
Cyclic loading of column compression force.

The compression force of the left-side column of the first story was monitored to examine the effect of the damper. Fig 17 shows the variation of the column force of frame models subjected to cyclic loading. The results of the damper-equipped frame resemble those of the BF because of the identical framing properties, indicating that the seismic retrofit using the proposed system only slightly influences the outer column members. Column buckling is a mode of failure which generally results from structural instability due to the compressive action on the column. The typical CB affects the column force in an inefficient manner which increases the risk of plasticity on the section that causes column buckling. Furthermore, the results show that a high axial force is induced in the braces, the peak axial forces due to cyclic loading are, on average, 3.5 and 5 times greater than those of the damper-equipped and bare frames, respectively.

## Conclusion

A new VE energy dissipation device was introduced to protect structures against lateral movements. This damping device uses readily accessible materials that can be installed in structures. The performance of the rubber material was evaluated through an experimental test and FE analysis. The VEM is considered to have a fine energy dissipation capacity as indicated by the smooth, plump ellipses of the hysteresis curves. Increased excitation rates caused an increased angle of inclination of the hysteresis with fixed displacement amplitude; this indicates that VEM stiffness was positively correlated with excitation rate. There was also a corresponding increase in the areas of the hysteresis curves that represented the energy dissipation capacity. Moreover, an increase in displacement amplitude resulted in an angle decrease and a larger hysteresis loop. The damper was installed at CB. It included of a number of steel plates and vertical rubber layers for providing a shear viscoelastic behavior after a dynamic excitation such as earthquake. The amount of energy dissipation of the RRBD was disclosed to be desirable through FEA and detailed parametric studies. Analyses resulted in recommendation of proper ranges of parameters for design purposes of the introduced damper. The seismic response of the frame provided with RRBD was investigated experimentally. The responses were compared with the BF in order to establish effectiveness of using RRBDs. It was observed that the displacement responses reduced significantly and the acceleration responses increased slightly owing to higher stiffness and damping. Moreover, cyclic analyses were performed for three-story steel moment frames to examine the effects of the proposed damper on columns. The axial load variations in the columns were resemble between the BF and the damper system. This resemblance indicates that the RRBD slightly influences the outer columns.

The proposed damper is inexpensive, easy to build, easy to install, and highly efficient. To conduct a comprehensive performance evaluation and to monitor the efficiency level of the damper with respect to the structural behavior, other building typologies such as different building heights, number of bays, and span lengths may need to be considered.
